# SmartFire: Intelligent Platform for Monitoring Fire Extinguishers and Their Building Environment

**DOI:** 10.3390/s19102390

**Published:** 2019-05-25

**Authors:** Roberto Garcia-Martin, Alfonso González-Briones, Juan M. Corchado

**Affiliations:** 1 Mechanical Engineering Department, University of Salamanca, 49022 Zamora, Spain; 2 BISITE Research Group, University of Salamanca, Edificio I+D+i, Calle Espejo 2, 37007 Salamanca, Spain; corchado@usal.es; 3 Air Institute, IoT Digital Innovation Hub (Spain), Carbajosa de la Sagrada, 37188 Salamanca, Spain; 4 Department of Electronics, Information and Communication, Faculty of Engineering, Osaka Institute of Technology, Osaka 535-8585, Japan; 5 Pusat Komputeran dan Informatik, Universiti Malaysia Kelantan, Karung Berkunci 36, Pengkaan Chepa, Kota Bharu 16100, Kelantan, Malaysia

**Keywords:** fire extinguishers, monitoring, SmartFire, soft agents systems

## Abstract

Due to fire protection regulations, a minimum number of fire extinguishers must be available depending on the surface area of each building, industrial establishment or workplace. There is also a set of rules that establish where the fire extinguisher should be placed: always close to the points that are most likely to be affected by a fire and where they are visible and accessible for use. Fire extinguishers are pressure devices, which means that they require maintenance operations that ensure they will function properly in the case of a fire. The purpose of manual and periodic fire extinguisher checks is to verify that their labeling, installation and condition comply with the standards. Security seals, inscriptions, hose and other seals are thoroughly checked. The state of charge (weight and pressure) of the extinguisher, the bottle of propellant gas (if available), and the state of all mechanical parts (nozzle, valves, hose, etc.) are also checked. To ensure greater safety and reduce the economic costs associated with maintaining fire extinguishers, it is necessary to develop a system that allows monitoring of their status. One of the advantages of monitoring fire extinguishers is that it will be possible to understand what external factors affect them (for example, temperature or humidity) and how they do so. For this reason, this article presents a system of soft agents that monitors the state of the extinguishers, collects a history of the state of the extinguisher and environmental factors and sends notifications if any parameter is not within the range of normal values.The results rendered by the SmartFire prototype indicate that its accuracy in calculating pressure changes is equivalent to that of a specific data acquisition system (DAS). The comparative study of the two curves (SmartFire and DAS) shows that the average error between the two curves is negligible: 8% in low pressure measurements (up to 3 bar) and 0.3% in high pressure (above 3 bar).

## 1. Introduction

One of the most crucial safety aspects of any building is ensuring that all the necessary fire safety measures are in place. The building must have a clearly marked emergency exit route that will safely guide people out of the building [1]. However, within the possibilities of the building, other measures must also be in place to try, as far as possible, to extinguish or contain fire. Fire extinguishers are one such measure, which are used to extinguish or control small fires in emergency situations. However, they are not designed for use in uncontrolled fires that endanger the user (i.e., no exit route, smoke, explosion hazard, etc.), or that requires the expertise of a fire department.

Typically, a fire extinguisher consists of a cylindrical pressure vessel containing an agent that can be discharged to extinguish fire. There are also fire extinguishers made from non-cylindrical pressure vessels but they are less common [2,3]. There are two main types of fire extinguishers: stored-pressure and cartridge-operated. In stored pressure units, the expellant is stored in the same chamber as the firefighting agent itself. Different extinguishers are used depending on the fuel that has caused the fire; these are classified by letters, which refer to the type of fire the extinguisher is designed for. The types of fire are:

- Class A: Fires that involve solid fuels such as wood, cardboard, plastic, etc.
- Class B: Fires that involve liquid fuels such as oil, gasoline or paint.
- Class C: Fires that involve gas fuels such as butane, propane or city gas.
- Class D: Fires of this type are the rarest, the fuel is a metal, and the burning metals are magnesium, sodium or aluminium powder.

Taking this categorization into account, we can better understand the types of available fire extinguishers and the fire extinguishing agents they contain. The most common extinguishing agents include:

- Water: Suitable for Type A fires always in places where there is no electricity. Water is not suitable for fires involving liquid fuels such as gasoline or oil because it is denser than those liquids; as a result, the fuel would remain on top of the water and it would not be possible to extinguish the fire.
- Water spray: Ideal for extinguishing Type A fires and suitable for Type B fires. They should never be used in the presence of electric current as water could cause electrocution. This type of fire extinguisher is good outside of homes where there is no electrical risk, such as gardens, barbecues, etc.
- Foam: Ideal for Type A and B fires; we have all seen firefighters spray foam at emergency drills. As with the previous one, it is dangerous in the presence of electricity.
- Dust: It is the most common type and is used in any building. It is suitable for fires of Types A, B and C. It is powdery so it can be used in the presence of electricity. It is the most recommended extinguisher for houses, offices or any other type of building.
- CO${}_{2}$ extinguishers: CO${}_{2}$ is a gas and thus cannot conduct electricity. This type of fire extinguisher is suitable for fires of Types A, B and C. It is usually used in the presence of delicate elements where other types of extinguishers would damage the objects. If we use a standard fire extinguisher in a laboratory, for example, the foam or powder could damage expensive machines and equipment. Thus, CO${}_{2}$ extinguishers are ideal for this type of environments. Although they are the most versatile of all available extinguishers, they are also the most sensitive ones; changes in temperature can affect them considerably. Given that they do not have a manometer, it is impossible to know their pressure in real time, thus manual inspection is necessary.

Since fire extinguishers are pressure appliances, they require maintenance operations to ensure they function properly in the case of a fire. For this reason, the condition of fire extinguishers is checked periodically, including their security seals, inscriptions, hoses, etc. This mainly includes checking the state of charge of the extinguisher (weight and pressure), the bottle of propellant gas (if available), and the state of all mechanical parts (nozzle, valves, hose, etc.). All this is done to make sure they are safe and that they will be effective in extinguishing a fire. Moreover, it is necessary to ensure that they are located in a visible place that is easy to access [4].

Currently, the inspection of the fire extinguishers must be performed by a specialized operator. These checks are carried out manually every three months, one year or five years, depending on the type of the fire extinguisher. All fire extinguishers cannot exceed twenty years of useful life. Thus, the investigation of automated inspection methods would be of great benefit as it would allow one to control the condition of the fire extinguishers in real time. As a result, the company responsible for inspecting the fire extinguishers would be notified immediately after an abnormal condition (in terms of weight or pressure) is detected by the automated system. Hence, an automated system is going to provide the confidence that all the fire extinguishers are in their optimal condition in the case of a fire. Moreover, this would contribute to economic saving since it would no longer be necessary for operators to go for periodic inspections. The automated system would review the state of all the fire extinguishers simultaneously. At the time of inspection, both mechanical components and pressure are checked. It is unusual to detect faults in the state of the mechanical components, although it is possible to detect faults in the state of the pressure of the extinguisher. The reason for inspecting both aspects of the fire extinguishers at the same time is more for economic reasons rather than functional reasons. However, reviews are also carried out in the event of detection of a mechanical defect (lack of any component, damaged elements, etc.) or variation in pressure (small and inaccurate manometers or lack thereof, e.g., in CO${}_{2}$ extinguishers).

To be able to monitor the state of the extinguishers in real time and perform autonomous control, it is necessary to place sensors on them, including pressure, temperature, humidity and smoke sensors [5,6]. Devices with high levels of pressure such as CO${}_{2}$ units require sensors that are more expensive than the extinguisher itself, and thus the proposed system provides a much more economical solution.

In this way, it is possible to analyze the impact of the incidence of factors such as humidity or temperature on the condition of the fire extinguisher. However, there are even greater possibilities if a position sensor is deployed. If the set of sensors includes a position sensor, it would be possible to find out (when the temperature began to rise in the building) when each fire extinguisher was used; how the fire spread through the building; and the order in which the fire extinguishers were used (when it was remove from its support). The platform will therefore provide mechanisms that will allow one to understand how people behave in the event of a fire. This, in turn, would allow one to assess the security measures deployed in the building and adapt them to the behavior of people in emergency situations [7,8].

This information is very useful as it enables us to understand how people act in the event of a fire. Human behavior during the initial phase of a fire is, therefore, an important factor in terms of survival [9,10]. This would enable the development or optimization of exit routes within buildings or dwellings.

The goal of the work presented in this article was to provide a novel system of soft agents for the detection of pressure changes in fire extinguishers, as well as to record changes in the parameters that affect them, such as temperature, humidity, etc. If the information indicates any incidence or if any value is outside its normal range of values, an alert will be sent [11]. There is a similar system called en-Gauge Fire Extinguisher (http://www.engaugeinc.net/fire-extinguisher-monitoring) to the one presented in this article but it is a commercial product adapted to a concrete fire extinguisher model, which warns when the pressure level has fallen below an operable level, and also requires the installation of a plate on the wall. Our prototype can be used in any fire extinguisher, does not require work on the wall and thanks to the web platform can visualize the pressure at any time, which will allow in the future including machine learning techniques to perform predictive maintenance processes.

The main contributions of this paper include: the use of sensors to acquire information on the state of fire extinguishers and the detection of anomalies in the pressure of the extinguisher without the need for human intervention. The platform will could even provides knowledge on the behavior of people during a fire and on their use of the fire extinguishers.This is because one will know the area where the fire originally occurred by knowing which extinguisher was used first. To complement the data acquired from the fire extinguisher, the developed platform obtains data from the building environment [12]. The developed system uses a combination of information from the fire extinguishers and the building environment, providing knowledge of variables that influence the state of fire extinguishers, making real-time inspection possible without the need for operators.

The rest of the article is structured as follows: Section 2 reviews related state-of-the-art projects and most commonly used technologies. Section 3 describes the SmartFire platform. Section 4 outlines the case studies that were performed to evaluate the proposed platform. Section 5 outlines the results. Finally, Section 6 draws conclusions from this proposal and discusses future lines of research.

## 2. Related Work

This section presents a thorough review of state-of-the-art literature in the field of fire extinguishers. We analyzed the variables that affect the pressure under which the internal composition of fire extinguishers is stored and also performed an in-depth study of how to correctly measure the pressure of a fire extinguisher from its outside. Moreover, we looked into different technologies used in the literature, examining their advantages and disadvantages for the development of our platform.

We must point out, however, that the current literature does not present any type of platform that would be capable of analyzing the parameters that affect the pressure in fire extinguishers. This makes evident the contribution of our platform, which collects data autonomously and controls whether the parameters of all fire extinguishers are similar, detecting and notifying possible incidents.

The measurement of pressure is related to the measurement of deformation and in the case of small deformations the technology most used for this purpose is the use of strain gauges. These are used fundamentally for the determination of tensions, such that in our case it is sought to relate these with the pressure [13,14].

Another of the conditioning factors/limitations of this work consisted in finding a low-cost monitoring platform that would not make the final price of the extinguisher more expensive, capable of taking measurements (temperature, humidity, etc.) inside buildings with wireless communication and low consumption [15].

### 2.1. Proposals with SmartFire Sub-Objectives

There are no systems that realize in a single prototype the objectives proposed in this article. Here are some works that have proposed some solutions for partial objectives covered by the SmartFire prototype. Park et al. proposed the measurement of pressure gauge using color segmentation for the safety management of fire extinguisher [16]. The main idea is that pressure gauge includes a green color indicating the normal pressure. In the work done by Jia-ming Jin, the release characteristics of the gas extinguishing agent from fire extinguisher vessel at different filling conditions was studied [17,18]. The results show that the outlet pressures of the fire extinguisher vessel have the same trend basically at different initial temperature: all of them decline rapidly with the jetting time and then tend to be gentle. Park et al. [18] proposed again a fire extinguisher maintenance system using smart NFC communication as well as real-time pressure measurement. The proposed system consists of three steps in the flow of information. The first step is to identify the fire extinguisher through NFC tagging in the fire extinguisher module using the smart device. The fire extinguisher appearance check and the real-time pressure measurement is performed in the second step, and the last step sends the check status information to the management server. In particular, the actual pressure value is calculated based on the angle of the green area and the indicating needle. However, the use of NFC severely limits these systems in large multi-story office buildings. Other work is based on measuring pressure drops such as the work presented by [19]. They developed an application of the portable fire extinguishing equipment in fire prevention of power transmission line results and showed that massive wildfires can be extinguished quickly. By using the equipment, electric power company’s ability of resisting wildfires has gained remarkable improvement.

### 2.2. Algorithm for Calculating the Pressure of a Fire Extinguisher

In mechanical design, to be able to calculate the stress of an element, it is necessary to know what material it is made of, its geometry and the conditions it is subjected to. In terms of the development of behavioral theories (the development presented in this paper), they are based on two principles: (i) equilibrium, i.e., both external forces (in this case pressure) and internal forces (the reaction of the material) counteract each other; and (ii) compatibility of deformations, i.e., the deformations in different elements are contiguous with each other.

With regard to pressurized vessels, there are two theories [20], depending on the relationship between the radius of the element and its thickness.

If the thickness (*t*) is at least one order of magnitude smaller than the inner radius, it will be considered as a thin-walled case; there are also some other upper limits for these criteria given in the literature such as $r_{i}/t>20$ where $r_{i}$ is the inner radius, which means that, when $r_{i}/t<20$, the thickness is more relevant than the radius [21]. Thus, a different formulation must be used in each case.

**Figure 1:**
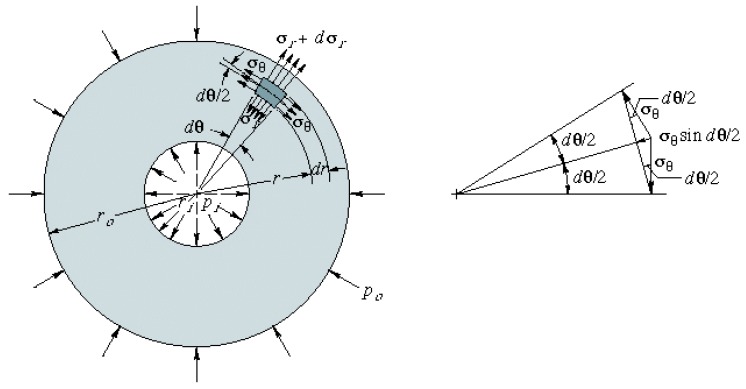
Stress diagram: thick-walled case.

A pressure vessel can be spherical or cylindrical with hemispheric or ellipsoidal endings. We took measurements of the cylindrical part since it is a well-defined area, in which the following stresses appear (Figure 1):

- $\sigma_{\theta }$, the tangential tension, goes in the tangent direction to the circumference. It is the highest of all and increases the perimeter of the cylinder, as a result of the level of deformations, the wall of the cylinder separates along its generator.
- $\sigma_{r}$, the radial stress goes in the direction of the radius at the deformation level, making the radius increase in length. It is the smallest of them all.
- $\sigma_{z}$, the stress in the direction z makes the cylinder increase in length, at the deformation level it is as if the cylinder were pulled at the ends and stretched.

In mechanical engineering, depending on the case, i.e., thin or thick wall, different formulations are available, the latter being the most complex.

**Figure 2:**
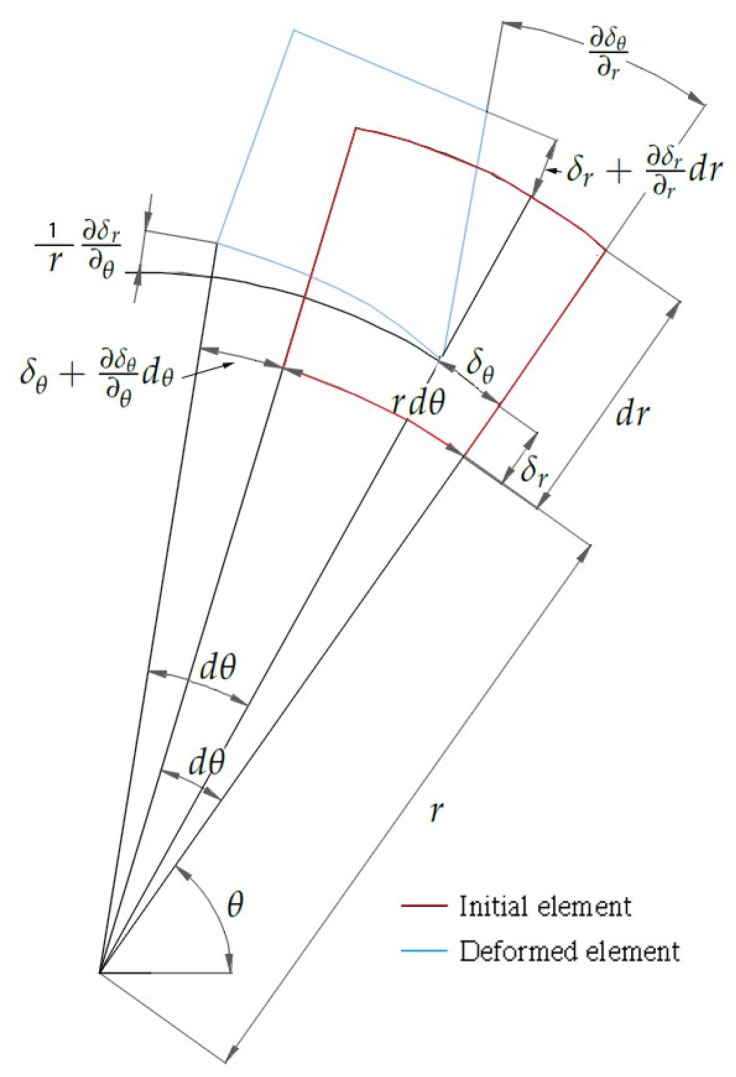
Deformation scheme: thick-walled case.

1. Thin wall, $r_{i}/t>20$. In this case, the effect of the thickness is not considered, hence the radial stress ($\sigma_{r}$) is not taken into account, since it is assumed that the tangential stress ($\sigma_{\theta }$) is much larger than the radial stress, which is therefore ignored; the expressions for these stresses [20] are:
  Tangential stress:
  (1) $$\sigma_{\theta }=Pr/t$$
  Axial stress:
  (2) $$\sigma_{z}=Pr/2t$$
  Radial stress:
  (3) $$\sigma_{r}=0$$
  These expressions relate internal pressure (P), radius (r) and thickness (t). For the case of the radius, we assume (since thickness is not considered) that both internal and external radii are equal ($r_{i}=r_{o}$). Some authors use the mean radius ($r_{m}$), which is more accurate.
  In our case, what we can measure are deformations and not stresses, but through Hooke’s Law [22], which relates tensions and deformations (Equations (5) and (6)) within the linear elastic regime, it is possible to relate the deformation caused by the internal pressure of the gauge because our extinguisher works in this regime.
  (4) $$\sigma_{ij}=E\varepsilon_{ij}$$
  The linear modulus of elasticity (E) is a known characteristic of the material, and $\varepsilon_{ij}$ is the deformation measured with the gauge, where the subscripts *ij* indicate the direction in which the deformation occurs.
  In radial systems, the spatial directions x,y,z are replaced by the radial direction (r), tangential (θ) and z is maintained.
  The objective of this work was to relate the deformation (ε) with the pressure (P) that it produces; we can relate the values of P with ε since we know the stress values as functions of the pressure (Equations (1) and (2)) and the value of the deformation (ε) is obtained directly from the gauge. Depending on how the gauge is positioned on the container, it is measured. If the gauge is positioned in the axial direction, the value of $\varepsilon_{z}$ is measured and if it is placed tangentially, $\varepsilon_{\theta }$ is obtained.
  (5) $$\sigma_{r,\theta ,z}=E\varepsilon_{r,\theta ,z}$$
  Then the value of the tangential stress (Equation (1)) is introduced in Equation (5); thus, via algebra, we can obtain the internal pressure of the vessel (Equation (6)) since it presents the relation P vs. $\varepsilon_{\theta }$ according to known geometrical parameters and material properties, in this case, the Young’s modulus (E), and the Poisson’s ratio (ν), which is a parameter of the material and is provided by the manufacturer or obtained through mechanical test. We operate in the same way to obtain the relationship between P and $\varepsilon_{z}$ (Equation (7)). In thin wall cases, the radial direction deformation value is ignored, and the gauge cannot be placed in this direction.
  A large diameter fire extinguisher with a common pressurization (13 bar) is a thin-walled type of fire extinguisher (wheeled extinguisher and extinguishing tanks). In Equation (5), the values measured by the gauge in tangential direction together with Equation (1) are replaced by Equation (6). Similarly, we operate in the z (axial) direction, providing Equation (7), which is the expression to be used in metal containers, since in the radial direction the deformation value is ignored and the gauge cannot be placed.
  Measurement of the deformation in tangential direction:
  (6) $$P=\varepsilon_{\theta }E\frac{t}{r_{m}}$$
  Measurement of axial deformation:
  (7) $$P=\varepsilon_{z}E\frac{2t}{r_{m}}$$
  Most of the portable devices are pressurized to 13 bar and their diameters are larger than the thickness (especially in wheeled extinguisher and extinguishing tanks) with the aim of optimizing the volume, thus thin wall is the most common situation, except the CO${}_{2}$ devices due to the high pressure.
2. Thick wall, $r_{i}/t<20$ In this case, the idea is the same, but the formulation is more complex because it is not possible to apply the simplification of negligible thickness in the equations, as it does have an influence here. In Figure 1, we see that the internal pressure generates a series of stresses that will deform the material, as shown in Figure 2. In Figure 2, we can see an undeformed and a deformed element, where the value of the deformation can be calculated by applying finite differences in such a way that we can obtain the value of the deformation according to the displacements.
  Equations (8) and (9) allow relating deformations to stress, and are fundamental equations exposed in the Fundamentals of Machine Elements deformation theory [23]. These equations are used in the following steps for the calculation of pressure.
  (8) $$\varepsilon_{r}=\frac{\delta_{r}+\frac{\partial \delta_{r}}{\partial_{r}}dr-\delta_{r}}{dr}=\frac{\partial \delta_{r}}{\partial_{r}}$$
  (9) $$\varepsilon_{\theta }=\frac{(r+\delta_{r})d\theta -rd\theta }{rd\theta }=\frac{\delta_{r}}{r}$$
  Here, again, our goal is to calculate pressure as a function of deformation. We know that pressure generates stresses that in turn generate deformations. Now, we reverse this logic and obtain the stresses from the deformations. This is achieved by applying Hooke’s law (Equation (4)) where the expressions of both radial (Equation (10)) and tangential (Equation (11)) deformations are functions of both radial and tangential stresses.
  (10) $$\varepsilon_{r}=\frac{1}{E}\left(\sigma_{r-\vartheta }\sigma_{\theta }\right)$$
  (11) $$\varepsilon_{\theta }=\frac{1}{E}\left(\sigma_{\theta -\vartheta }\sigma_{r}\right)$$
  By equating Equations (8) and (10), we obtain Equation (12), and, by equating Equations (9) and (11), we obtain Equation (13).
  (12) $$\frac{\partial \delta_{r}}{\partial r}=\frac{1}{E}\left(\sigma_{r-\vartheta }\sigma_{\theta }\right)$$
  (13) $$\frac{\delta_{r}}{r}=\frac{1}{E}\left(\sigma_{\theta -\vartheta }\sigma_{r}\right)$$
  Equations (12) and (13) form a system of two equations and three unknowns, where, if we apply the criterion of equilibrium to the system of forces that generate the tensions (Figure 1), we obtain Equation (14), such that this equation complements the previous system and it can be solved as a compatible and determined system.
  (14) $$\begin{array}{c} \left(\sigma_{r}+d\sigma_{r}\right)\left(r+dr\right)d\theta dz-\sigma_{r}r\theta dz-2\sigma_{\theta }\sin\frac{d\theta }{2}drdz=0\\ \sin\left(\frac{d\theta }{2}\right)=\frac{d\theta }{2}\to \sigma_{\theta }=r\frac{d\sigma_{r}}{dr}+\sigma_{r} \end{array}$$
  By substituting Equation (14) into Equation (13) and deriving it from r, we get Equation (15)
  (15) $$\begin{array}{c} \frac{\delta_{r}}{r}=\frac{1}{E}\left(r\frac{d\sigma_{r}}{d_{r}}+\sigma_{r-\vartheta }\sigma_{r}\right)\to \delta_{r}=\frac{r}{E}\left(r\frac{d\sigma_{r}}{d_{r}}+\sigma_{r-\vartheta }\sigma_{r}\right)\\ \frac{\partial \delta_{r}}{\partial r}=\frac{2r}{E}\frac{\partial \sigma_{r}}{\partial_{r}}+\frac{r^{2}}{E}\frac{\partial^{2}\sigma_{r}}{\partial r^{2}}+\frac{\sigma_{r}}{E}+\frac{r}{E}\frac{\partial \sigma_{r}}{\partial r}-\frac{\vartheta \sigma_{r}}{E}-\frac{r\vartheta }{E}\frac{\vartheta \sigma_{r}}{\vartheta_{r}} \end{array}$$
  If we now substitute Equation (14) into Equation (12) and equate it to Equation (15), by substituting and operating, we obtain the relationship in Equation (16), which can also be expressed as Equation (17).
  (16) $$0=3\frac{\partial \sigma_{r}}{\partial r}+r\frac{\partial^{2}\sigma_{r}}{\partial r^{2}}$$
  (17) $$0=2\frac{\partial \sigma_{r}}{\partial r}+\frac{\partial }{\partial r}\left(r\frac{\partial \sigma_{r}}{\partial r}\right)$$
  Integrating once:
  (18) $$0=2\sigma_{r}+r\frac{\partial \sigma_{r}}{\partial r}+C_{1}$$
  Re-integrating and simplifying:
  (19) $$0=\frac{\partial }{\partial r}\left(r^{2}\sigma_{r}\right)+C_{1}r$$
  Integrating once again:
  (20) $$\sigma_{r}=\frac{-c_{1}}{2}-\frac{-c_{2}}{r^{2}}$$
  Applying the boundary conditions to Equation (20), for a general pressurization case where we would have to:
  (21) $$\sigma_{r}{|}_{r=r_{i}}=P_{i}\to P_{i}=\frac{-c_{1}}{2}-\frac{-c_{2}}{r_{i}^{2}}$$
  (22) $$\sigma_{r}{|}_{r=r_{0}}=P_{o}\to P_{o}=\frac{-c_{1}}{2}-\frac{-c_{2}}{r_{o}^{2}}$$
  By operating, we obtain the value of the constants:
  (23) $$c_{2}=\left(\frac{P_{o}-P_{i}}{r_{o}-r_{i}}\right)\left(\frac{r_{o}^{2}-r_{i}^{2}}{r_{i}^{2}r_{i}^{2}}\right)$$
  (24) $$c_{1}=-2\left[\frac{1}{r_{o}^{2}}\left(P_{o}-P_{i}\right)\left(\frac{r_{o}^{2}-r_{i}^{2}}{r_{i}^{2}r_{i}^{2}}\right)+P_{o}\right]$$
  such that, by substituting the value of the constants in Equation (20) and simplifying, we obtain:
  (25) $$\sigma_{r}=\frac{P_{i}r_{i}^{2}-P_{o}r_{o}^{2}+\left(P_{o}-P_{i}\right){\left(\frac{r_{o}r_{i}}{r}\right)}^{2}}{r_{o}^{2}-r_{i}^{2}}$$
  The tangential stress is provided by Equation (14), where by substituting the value of the radial stress (Equation (25)) and the value of its derivative with respect to r (Equation (26))
  (26) $$\frac{\partial \sigma_{r}}{\partial_{r}}=\frac{-2\left(P_{o}-P_{i}\right){\left(r_{o}r_{i}\right)}^{2}}{r^{3}\left(r_{o}^{2}-r_{i}^{2}\right)}$$
  (27) $$\begin{array}{c} \sigma_{\theta }=r\frac{-2\left(P_{o}-P_{i}\right){\left(r_{o}r_{i}\right)}^{2}}{r^{3}\left(r_{o}^{2}-r_{i}^{2}\right)}+\frac{P_{i}r_{i}^{2}-P_{o}r_{o}^{2}+\left(P_{o}-P_{i}\right){\left(\frac{r_{o}r_{i}}{r}\right)}^{2}}{r_{o}^{2}-r_{i}^{2}}=\frac{P_{i}r_{i}^{2}-P_{o}r_{o}^{2}-\left(P_{o}-P_{i}\right){\left(\frac{r_{o}r_{i}}{r}\right)}^{2}}{r_{o}^{2}-r_{i}^{2}} \end{array}$$
  For this particular case, where $P_{o}$ << $P_{i}$, it is assumed that $P_{o}$ = 0, substituting in Equations (25) and (27) would give particular stress values.
  (28) $$\begin{array}{c} \sigma_{r}=\frac{P_{i}r_{i}^{2}\left(1-{\left(\frac{r_{o}^{2}}{r^{2}}\right)}^{2}\right)}{r_{o}^{2}-r_{i}^{2}}\\ \sigma_{\theta }=\frac{P_{i}r_{i}^{2}\left(1+{\left(\frac{r_{o}}{r}\right)}^{2}\right)}{r_{o}^{2}-r_{i}^{2}} \end{array}$$
  Finally, to return to Equation (11), we can relate the deformation with the pressure
  (29) $$\varepsilon_{\theta }=\frac{1}{E}\left(\sigma_{\theta -\vartheta }\sigma_{r}\right)=\frac{1}{E}\left(\frac{P_{i}r_{i}^{2}\left(1+{\left(\frac{r_{o}}{r}\right)}^{2}\right)}{r_{o}^{2}-r_{i}^{2}}-\vartheta \frac{P_{i}r_{i}^{2}\left(1-{\left(\frac{r_{o}}{r}\right)}^{2}\right)}{r_{o}^{2}-r_{i}^{2}}\right)$$
  Since the strain gauge is placed on the outside radius, the expression is reduced to:
  (30) $$\varepsilon_{\theta }=\frac{1}{E}\left(\frac{P_{i}r_{i}^{2}\left(1+\frac{r_{o}^{2}}{r_{o}^{2}}\right)}{r_{o}^{2}-r_{i}^{2}}-\vartheta \frac{P_{i}r_{i}^{2}\left(1-\frac{r_{o}^{2}}{r_{o}^{2}}\right)}{r_{o}^{2}-r_{i}^{2}}\right)=\frac{1}{E}\frac{2P_{i}r_{i}^{2}}{r_{o}^{2}-r_{i}^{2}}\to P_{i}=\varepsilon_{\theta }E\frac{r_{o}^{2}-r_{i}^{2}}{2r_{i}^{2}}$$
  Since both the material and the geometry are known, by placing a gauge on the outside of the fire extinguisher in the tangential (circumferential) direction, we can determine the value of the internal pressure in the vessel (fire extinguisher).

### 2.3. Agent Based Monitoring Platform

Nowadays, the evolution of the electronics field has allowed us to greatly reduce the size of sensors, thus we are able to collect values for very different parameters through the placement of small devices. The field of communications has also evolved enormously through the development of communication protocols that allow us to send long distance data with little power consumption. For this reason, the developed device is going to allow us to measure the pressure of the extinguishers autonomously and send the measurements to an external platform that manages those measurements and detects anomalies.

There is no device for measuring pressure changes in fire extinguishers with the size and accuracy characteristics required for this task, nor is there a platform to monitor a set of fire extinguishers and send notifications to the personnel responsible for their maintenance. There are some similar works but their focus is not the same as that of this work. One of these works is the one proposed by Chow, who presented a fire safety classification system (EB-FSRS) to evaluate the fire safety provisions in the existing high rise non-residential buildings in Hong Kong. The aim is to investigate to what extent the fire safety provisions of existing buildings deviate from the expectations of the new codes [24]. Other work has focused on the development of computer vision techniques to be used by drones to detect fires in open spaces, such as those developed by Chamoso et al. [25] and Verstock et al. [26].

Rashid et al. developed a multi-sensor-based fire-extinguishing robot and demonstrated its implementation with a brief discussion on its construction and operation [27]. As can be seen from these works, the great majority are focused on extinguishing or detecting fires but not on ensuring that the security measures are in optimum condition.

In this respect, the system of soft agents allows one to implement one agent in each SmartFire prototype in a building in a way that allows them to communicate, coordinate and cooperate when monitoring the set of extinguishers in the building. This agent methodology has been widely applied in the monitoring work in various areas [28,29].

Thanks to the monitoring of the environmental conditions of an extinguisher, the use of this methodology in the future is going to make it possible to analyze the origin of a fire, the conditions in which it occurred and the behavior of people in this situation. With the environmental values of each extinguisher, new soft agents can be deployed for the application of big data techniques based on preventing possible sources of fires.

## 3. SmartFire Platform

This section details the SmartFire architecture, the description of the prototype and the software architecture that allows us to apply the algorithm for calculating the pressure of a fire extinguisher.

### 3.1. Prototype Overview

**Figure 3:**
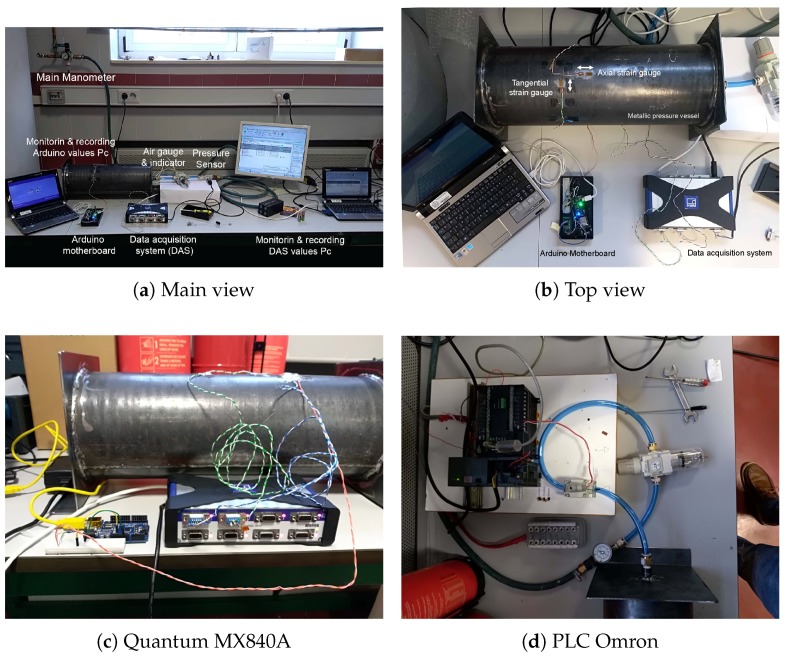
Overview of the pressure measurement equipment.

At the Mechanical Engineering Department laboratory, University of Salamanca, Zamora, we have HBM equipment, a Quantum MX840A (Figure 3c), which is used specifically for data acquisition and is particularly accurate when measuring with strain gauges. The Quantum system is used exclusively to calibrate the SmartFire prototype.

**Figure 4:**
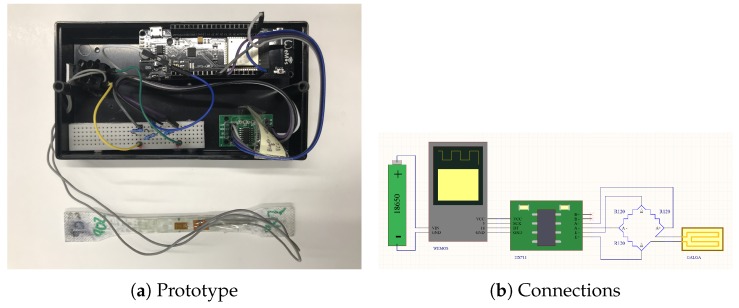
SmartFire prototype.

The proposed architecture (Figure 4) requires both a signal capture system (input) and wireless communications (output) all managed by a control system. The management system uses a microcontroller of the Arduino series, specifically the wemos WiFi and bluetooth Battery, which allows for wireless communications. This is of vital importance because the system should be coupled with the extinguisher and be an isolated component. In other words, the system does not need to be connected to a power supply network or to a fixed data network, as it has an autonomous power supply system (it can even be connected to a solar panel). The prototype includes a DH22 sensor on the outside of the housing to also obtain temperature and humidity data.

The features are: WiFi module (ESP-WROOM-02) with digital and analog 10-bit I/O, hence the use of the amplifier (HX711 Load Cell Amplifier Module), which provides 12 bits. The amplifier is the same as that used when weighing systems, which in the end uses a strain gauge that relates the deformation of the weighing systems with the applied load. The latter is connected to a wheatstone bridge for gauging.

A Programmable Logic Control (PLC) of the Omron CP1H-XA brand is used (the A indicates that it has an analog I/O card; Figure 3d). This element is used to control all the generated test equipment (general prototype). The PLC manages the entire power system of the installation, controls the valves of the circuit that provide the pressure to the test specimen (pressure vessel). In addition, the pressure sensor is connected to the analog board of the PLC, which works in trigger mode, sending the order to both, the prototype and the Quantum to record the pressure values obtained in steps of 0.5 bar (50 kPa).

Pneumatic elements: The laboratory of Mechanical Engineering of the EPSZ has diverse pneumatic material with which the tests were performed, from the compressor, ducts, and electrovalves to instruments for measuring pressure, both basic (manometers) and high precision (head pressure sensor rotary 0–10 mpa Panasonic), as well as pressure regulators. The manometers are used as redundant instruments (a total of two are used) to verify that the measurement offered by the PLC of the pressure sensor is within the proper range (the accuracy of the pressure gauge is ±0.1 bar and that of the sensor is ±0.01 bar. This is done as a control method, to avoid configuration failures in the PLC).

### 3.2. Soft Agent Platform

The autonomy of soft agent systems allows them to interact with each other without human intervention. Their ability to perceive and react to changes in the environment makes this methodology an ideal approach for obtaining environmental data and responding to those changes with appropriate actions. Characteristics such as extensibility and flexibility make it possible to add new functionalities or include other algorithms and sensors. These advantages have led to numerous monitoring proposals that employ agent systems [30,31].

Agent systems are often applied in the field of process automation due to their ability to deal with more complex systems. Various agent systems have been developed for the management of energy optimization processes [29]. However, there is no record of any state-of-the-art developments that would use a soft agent platform to monitor the state of fire extinguishers in a building. This soft agent platform allows us to communicate with the SmartFire prototype for the reception of pressure values in real time for each of the monitored fire extinguishers.

**Figure 5:**
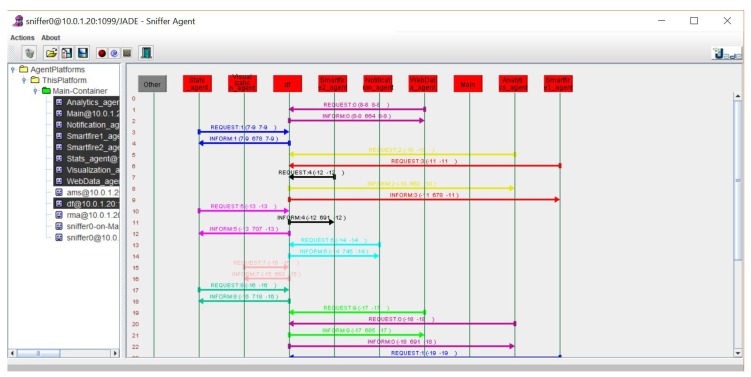
Visualization of agent communications on the SmartFire agent platform (JADE sniffer).

**Figure 6:**
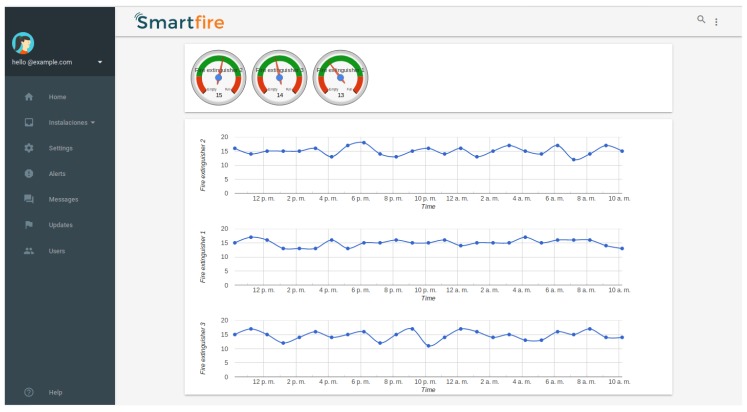
Visualization of the pressure values of the different extinguishers deployed in the building via the SmartFire web system.

**Figure 7:**
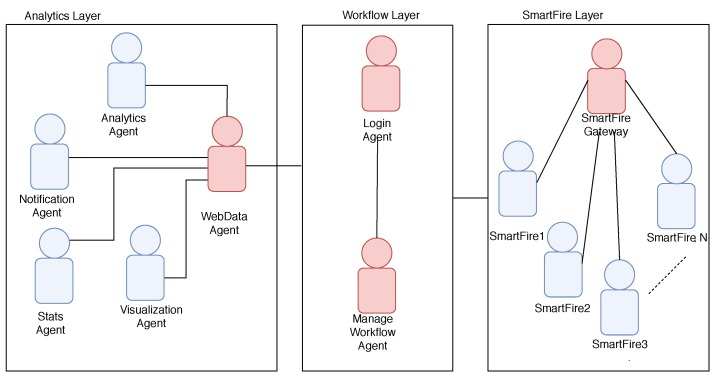
SmartFire platform diagram of the functional concept of the architecture.

The platform was developed using the JADE framework which facilitates the development of communication processes between agents by making use of the FIPA-ACL communication standard (Figure 5). This platform based on an architecture of lightweight agents communicates with the SmartFire prototype for the reception of pressure values in real time for each fire extinguisher monitored. The platform makes it possible to visualize this data through a website, as shown in Figure 6, and to send notifications to the people responsible for the maintenance of the extinguishers in the event that a fire extinguisher is out of the threshold of normal pressure values. Figure 7 shows the diagram of the functional concept of architecture, which shows how the agent-based system allows to carry out part of the reception of the measurements of the deployed prototypes to make notifications, statistics or visualization of data among others.

## 4. Case Study: Laboratory Validation

**Figure 8:**
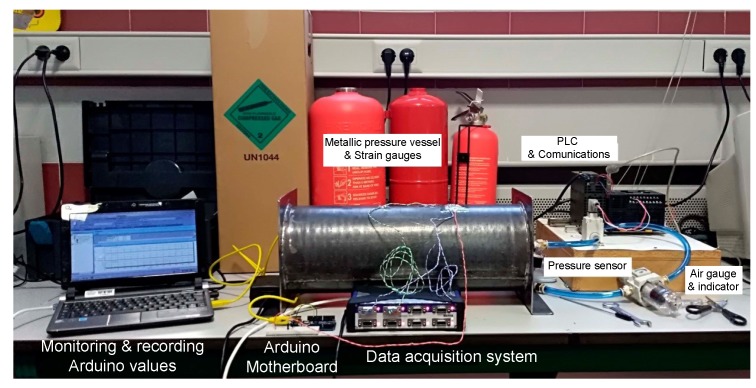
Laboratory validation of pressure measurement equipment.

This section details all the components that were involved in the case study (Figure 8) and how the case study was conducted.

The main idea was to take redundant measures and make sure that all of their values were the same, so that some methods would work for others. Step 1: Make a thin wall pressure vessel. Since the theory of cylinders has been sufficiently tested and its use for metallic materials has been proven, a calibrated specimen made of metal was constructed.

In our case, a controlled pattern pressure vessel (Figure 3 and Figure 8) was manufactured with the following dimensions: inner diameter, $d_{i}$ = 170 mm; length, 450 mm; and thickness, t = 1.5 mm. The vessel was made of steel of controlled properties (Table 1).

The dimensions of this test specimen (pressure vessel) were mainly determined by the pressure limitations of the test laboratory; although it was possible to access higher pressure levels, this was not necessary since the results were perfectly extrapolated.

**Figure 9:**
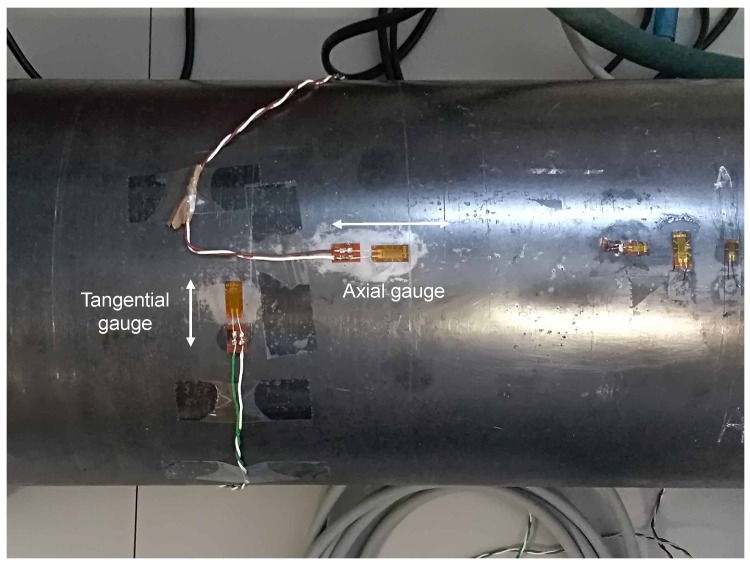
Strain gauges.

A series of strain gauges (Figure 9) were placed on the extinguisher, arranged in both radial and axial directions, the purpose of which was to capture measurements in different directions, since redundant measurements increase precision.

These gauges were connected to both of HBM’s commercial data acquisition system (DAS), Quantum (Figure 3c, blue and green cables), and the microcontroller (Figure 3, orange cable). The data acquired by the Quantum were displayed on the notebook (Figure 8) through the commercial Catman Easy software version 3.1.3.22.

Step 2: Calibrate pressure sensor. By means of the pressure regulator (Figure 3a), which includes a manometer, fixed pressure values were administered to the container. The values were verified with the different manometers inserted in the circuit and compared with the reading provided by the pressure sensor (Figure 3a). The pressure sensor was placed in a series with the rest of the manometers in the circuit. The reading of the pressure sensor was performed by the PLC, to which the pressure sensor was initially connected. This reading was displayed on the computer through its control software (CX-one), which provided measurements in real time. It was observed that the readings of all the pressure gauges, as one of the pressure sensors, coincided with what the calibration of the PLC considered good. The function of the PLC was to detect the pressure level to obtain the pressure values. Once the pressure set point was reached, it allowed 10 s to elapse. During this period, the value stabilized and was then recorded.

Step 3: Check the gauge dimension. Once the validity of the pressure measurement system was checked, the strain gauge measurement was checked and the formulation was applied (Section 2.2). The specimen parameters (material and geometry) and pressure were entered into the computer and the theoretical deformation measurement of the vessel was obtained in both tangential and axial directions. The theoretical results were then compared with the measurements provided by the Quantum. The obtained results confirmed the measures.

**Figure 10:**
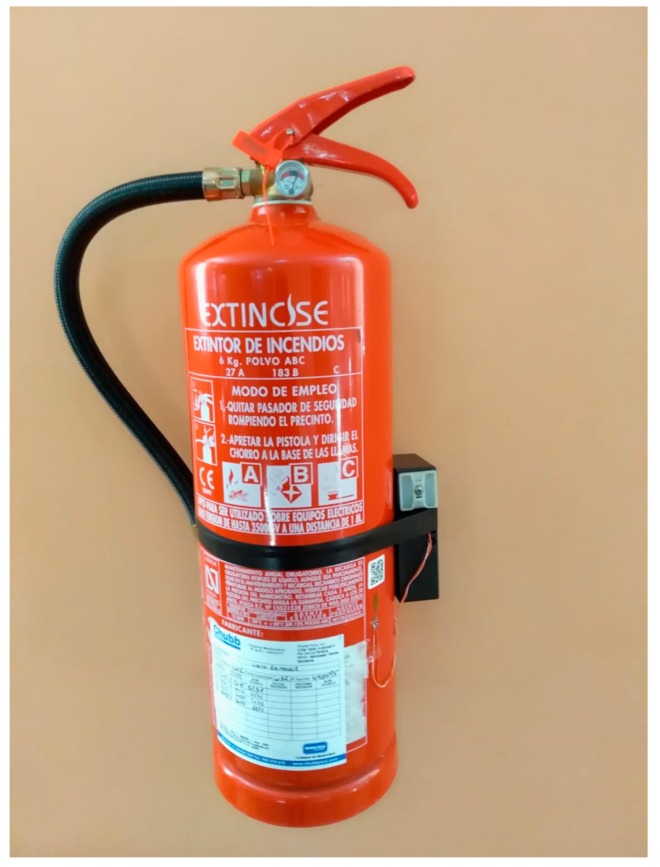
A SmartFire prototype situated in a fire extinguisher in the building of the case study.

Step 4: Check the SmartFire system measurement Figure 10. Pressure was applied progressively, where the PLC, at prescribed pressure steps, ordered both the Quantum and the SmartFire prototypes to register the deformation values, such that pressure–deformation measurements were obtained from both systems.

## 5. Results

**Figure 11:**
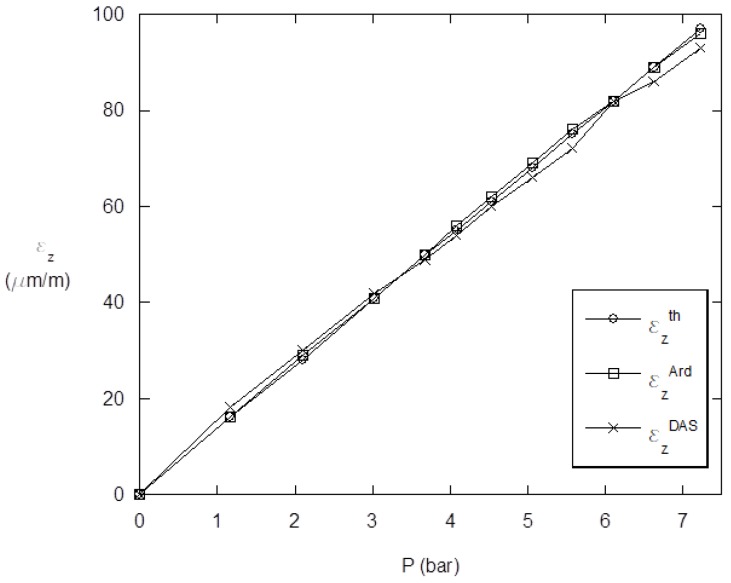
Comparison chart of the measurements obtained: $\varepsilon_{z}^{th}$, theoretical values; $\varepsilon_{z}^{Ard}$, SmartFire; $\varepsilon_{z}^{DAS}$, data Acquisition system. All values correspond to the longitudinal deformations ($\varepsilon_{z}$) included in Table 2, and are expressed in microns. Notice that the longitudinal axis is used as reference for the deformations, whereas the transversal axis is used for validating the DAS measurements.

Once the case study was validated, it was necessary to compare the results obtained by both the PLC system and the SmartFire prototype to validate whether the prototype was as precise in calculating the changes in pressure as the professional machine. To find out if the small prototype had the same precision, the curves obtained in Step 4 of the case study were compared (Figure 11).

This comparison showed that the error between the two curves occurred in low pressure measurements (up to 3 bars) was an average of 8%. At high pressure (from 3 bar up to 8 bar, maximum value used in the test), there was an average error of 0.3%, a negligible value. These values were used to verify the measurement capabilities of SmartFire.

Taking into account that the input data (geometry, material and pressure) are known, the theoretical value of deformation was calculated and then compared with the ones obtained experimentally (Table 2). According to the obtained results, the deviation between the data obtained by the prototype vs. theoretical values was always lower than 2%, whereas the values obtained by DAS vs. theoretical ones were lower than 4%. From these results, it can be concluded that the errors were higher in low pressure values in the testing program. This was predictable since the deformation was too low and the temperature affected the gauges.

## 6. Conclusions and Future Work

In this work, a novel platform has been presented for monitoring fire extinguishers in a building. The platform consists of a prototype that makes it possible to detect changes in pressure in real time and record the environmental conditions in which they have occurred (temperature, humidity, etc.). The use of soft agents has allowed for communication between the SmartFire prototype and the platform. Moreover, it has made it possible to read the pressure values of each extinguisher at all times. The architecture on which the platform is based allows us to integrate new agents by monitoring new factors, including agents that implement big data techniques to predict when a fire extinguisher will fail before it is produced. Experimental validation in the laboratory has allowed us to certify that the SmartFire prototype rigorously complies with the precision requirements of safety monitoring devices. In addition, the SmartFire prototype allows, in a very economical way, to carry out a review of the extinguishers of a building versus the traditional method, as it is not necessary to send anybody to check the device physically, because one can know instantaneously when the extinguisher ceases to be in optimal conditions.

As future lines of work, we intend to design a case study in an office building for real-time monitoring of fire extinguishers. This will allow us to find out when a fire extinguisher is not suitable for use immediately after an anomaly occurs. Moreover, it will be possible to calculate the average time that the extinguisher would continue to be in an anomalous state if only traditional inspections existed. This case study would show the average time that elapses from the time an anomaly has occurred in the fire extinguisher, to the moment it gets its periodic check, which can be up to three months, a period by which many extinguishers may have failed. Carrying out this experiment will further validate this proposal, eliminating high-risk situations in which there is a fire in a building whose fire extinguishers are not operational. Furthermore, The case of CO${}_{2}$ extinguishers will be studied in more detail, so much so that in this study it has become clear that it is a different and more complex case that requires a more in-depth study.

## Figures and Tables

**Table 1:** Characteristics of steel St 37-2 according to standard EN 10025-2: 2004, grade S235JR.

Property	Value	Unit
**Mechanical**		
Ultimate Strength	425	MPa
Yield Strength	333	MPa
Young’s modulus	205	GPa
Poisson’s ratio	0.29	–
**Thermal**		
Thermal expansion coefficient	11.5	μstrain/°C

**Table 2:** Longitudinal deformation measurement (in microns) by different techniques vs. Pressure. $\varepsilon_{z}^{th}$, theoretical values; $\varepsilon_{z}^{Ard}$, SmartFire; $\varepsilon_{z}^{DAS}$, data Acquisition system.

Pressure (Bar)	$\varepsilon_{z}^{\mathrm{th}}$	$\varepsilon_{z}^{\mathrm{Ard}}$	$\varepsilon_{z}^{\mathrm{DAS}}$
0	0	0	0
1.17	16	16	18
2.097	28	29	30
3.02	41	41	42
3.677	50	50	49
4.072	55	56	54
4.532	61	62	60
5.056	68	69	66
5.575	75	76	72
6.105	82	82	82
6.63	89	89	86
7.226	97	96	93
